# A Chatbot to Support Young People During the COVID-19 Pandemic in New Zealand: Evaluation of the Real-World Rollout of an Open Trial

**DOI:** 10.2196/38743

**Published:** 2022-11-04

**Authors:** Nicola Ludin, Chester Holt-Quick, Sarah Hopkins, Karolina Stasiak, Sarah Hetrick, Jim Warren, Tania Cargo

**Affiliations:** 1 Department of Psychological Medicine The University of Auckland Auckland New Zealand; 2 School of Computer Science The University of Auckland Auckland New Zealand; 3 Centre for Youth Mental Health University of Melbourne Melbourne Australia

**Keywords:** COVID-19, youth, chatbots, adolescent mental health, dialog-based intervention, digital mental health

## Abstract

**Background:**

The number of young people in New Zealand (Aotearoa) who experience mental health challenges is increasing. As those in Aotearoa went into the initial COVID-19 lockdown, an ongoing digital mental health project was adapted and underwent rapid content authoring to create the Aroha chatbot. This dynamic digital support was designed with and for young people to help manage pandemic-related worry.

**Objective:**

Aroha was developed to provide practical evidence-based tools for anxiety management using cognitive behavioral therapy and positive psychology. The chatbot included practical ideas to maintain social and cultural connection, and to stay active and well.

**Methods:**

Stay-at-home orders under Aotearoa’s lockdown commenced on March 20, 2020. By leveraging previously developed chatbot technology and broader existing online trial infrastructure, the Aroha chatbot was launched promptly on April 7, 2020. Dissemination of the chatbot for an open trial was via a URL, and feedback on the experience of the lockdown and the experience of Aroha was gathered via online questionnaires and a focus group, and from community members.

**Results:**

In the 2 weeks following the launch of the chatbot, there were 393 registrations, and 238 users logged into the chatbot, of whom 127 were in the target age range (13-24 years). Feedback guided iterative and responsive content authoring to suit the dynamic situation and motivated engineering to dynamically detect and react to a range of conversational intents.

**Conclusions:**

The experience of the implementation of the Aroha chatbot highlights the feasibility of providing timely event-specific digital mental health support and the technology requirements for a flexible and enabling chatbot architectural framework.

## Introduction

### Background

Looking after young people’s mental health is a global public health priority [1]. In New Zealand (Aotearoa), the rates of mental health challenges are increasing among rangatahi (young people) [2]. There are a range of supports being implemented nationally at present to transform care for young people and improve mental health. The Aroha chatbot is one such support tool.

The COVID-19 global pandemic has created trying circumstances at the population level around the globe because of the unprecedented changes and ongoing uncertainty about the future [3]. The COVID-19 pandemic has ongoing impacts on young people in terms of the transition from education to vocation [4-6], and in the context of developmental changes and emergence to adulthood [1].

Before the onset of the pandemic, it was estimated that between 10% and 20% of children and young people experience mental health challenges. This represents 60% to 70% of disability-adjusted life years among young people [7]. Despite many young people needing support and most not seeking or receiving any mental health care, public mental health services are organized in a way that can create significant barriers for those who do seek care. This includes limited capacity, even for those with more severe needs, lack of convenience or visibility, and cost. Other barriers include services being unacceptable to young people with fears such as lack of confidentiality, lack of privacy, and stigma. There is also a lack of equity of access to services [8].

There is now a large and growing body of research on the use of digital mental health support for young people. Digital tools have the benefits of being nonjudgemental, private, stigma free, flexible, and accessible, and have far greater reach than traditional forms of treatment. Digital mental health tools have been shown to be acceptable and effective treatments for the common mental health problems of anxiety and depression in youth [9,10].

### Conversational Agents

A dialog agent or “chatbot” style interaction for digital mental health has attracted interest since Eliza in the 1960s [11], even though its imitation of a psychotherapist through the simple linguistic token manipulation that was possible at the time was limited. A contemporary example of a conversational agent is Woebot, which delivers cognitive behavioral therapy (CBT). When tested with students with depression, those who used Woebot significantly reduced their symptoms over the study period. There was no reduction in symptoms for those in the information control group, who were offered a self-help book [12]. A systematic review of conversational agents in health care found mental health to be the most common area of application [13].

### COVID-19 Pandemic in Aotearoa

On March 21, 2020, at the beginning of the COVID-19 pandemic in Aotearoa, a 4-level COVID-19 alert system was announced [14]. Beginning at 11:59 PM on March 25, 2020, alert level 4 was instituted, putting the country into a nationwide lockdown with strict stay-at-home orders. This lockdown remained in place until 11:59 PM on April 27, 2020, and then was withdrawn in stages to the lowest alert level on June 8, 2020. With the perception that young people would benefit from and enjoy tailored digital support, we saw an opportunity to leverage existing digital infrastructure to guide rangatahi in Aotearoa through the stringent requirements of lockdown.

This period represented an opportunity for the uptake and trial of digital mental health technologies, and we used the Aroha chatbot. There was an apparent requirement for support other than face-to-face support, as community access was largely suspended and demand for support increased during this time [15].

### Responsiveness to Tāngata Whenua

Our research is grounded in Te Tiriti O Waitangi (the Treaty of Waitangi). Māori as Tāngata Whenua (the indigenous people of Aotearoa) have their indigenous status supported through government legislation in Te Tiriti O Waitangi, which guarantees partnership, participation, and protection for Māori. These principles are critical as we try to address the inequities that exist for Māori in a range of health outcomes in Aotearoa [16-18].

The HABITs (Health Advances through Behavioural Intervention Technologies) project has been developing an ecosystem of screening and e-therapy tools designed to meet the needs of young people in Aotearoa since 2016. The Aroha chatbot was developed out of the HABITs project, and the project was greatly inspired by the effectiveness and approach of SPARX (Smart, Positive, Active, Realistic, X-factor thoughts), which is an online game [19]. It helps young people who are feeling down (depressed, stressed, anxious, and low). The SPARX program’s development included a Māori cocreator, input from Māori CBT experts, cultural guidance from kaumātua (respected elder), and a Māori game development company [20]. The Māori and Pākehā (New Zealander of European descent) co-leadership in the HABITs project is crucial. It has ensured that as a design team, we are transformational and support the indigenization of mental health services for Māori [21].

Working biculturally (Māori and Pākehā) guaranteed not only relevant content, but also a respectful process in the co-creation of the Aroha chatbot. For this chatbot to connect with rangatahi Māori, we took guidance from our Māori advisors and chose Aroha as a name. Aroha means caring and kind; this allowed for Aro (meaning focus) and Hā (essence) to be included in the intent of the application, which was to provide a “caring and kind” e-therapy support tool that “focuses” on a person’s “essence.” In addition, strategies and activities were designed for and targeted rangatahi Māori and their whānau (family).

### Objectives

The objective of this study was to support young people in Aotearoa in managing challenges during the COVID-19 lockdown. We sought to develop chatbot architecture by leveraging existing technology, determine the process and experience of developing the Aroha chatbot, and evaluate the real-world rollout of an open trial.

## Methods

### Initial Architecture

Existing chatbot technology and broader online trial infrastructure already developed for the HABITs project were leveraged to develop the Aroha chatbot. The base chatbot technology for content management and delivery in the HABITs project was developed in partnership with a contractor, RUSH Digital [22]. This technology had already been used for chatbot deployments called “Headstrong” and “Stress-Detox.” Each of these were designed to promote resilience with methods grounded in CBT and positive psychology, and field trials have been conducted for each [22,23].

**Figure 1:**
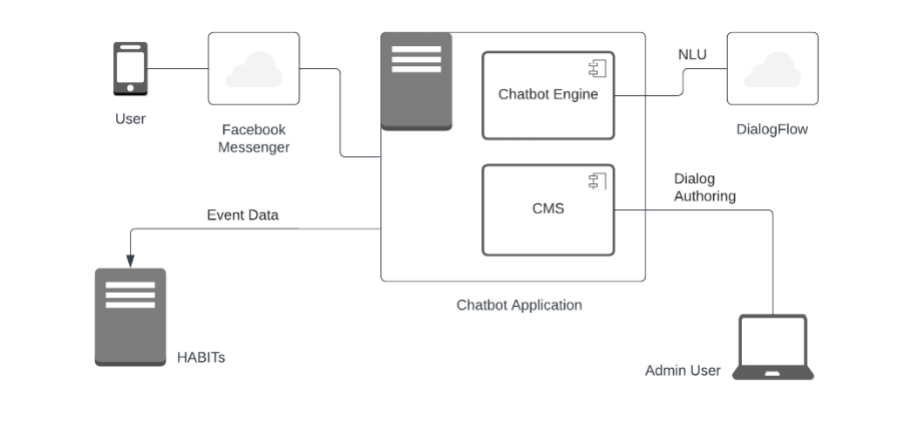
Simplified hybrid component architecture diagram of the system used for Aroha. CMS: content management system; HABITs: Health Advances through Behavioural Intervention Technologies; NLU: natural language understanding.

A high-level overview of the chatbot architecture is depicted in Figure 1. The main components are the messaging channel, chatbot engine, natural language understanding module, and dialog content authoring system (content management system [CMS]). The web application framework Django was used for the application technology, in combination with Facebook Messenger as a client.

The messaging channel selected for Aroha was Facebook Messenger, the sole channel initially supported by the base technology. Facebook Messenger (now Messenger by Meta Platforms) supports sending text with or without quick reply options; a variety of attachment types including simple and carousel images, GIFs, audio, and video; and buttons and webviews (web pages that display within Messenger). For instance, webviews were used in Aroha for a set of games called “Swipe Sports.”

The chatbot engine handled the user input/chatbot response conversational exchange. User input in the messaging channel invokes a request to the chatbot server, where chat requests are handled by the chatbot engine, dispatching one or more responses back to the user. In this fashion, the user has the experience of chatting with a persona (in this case Aroha) on Messenger. Aroha appears alongside contacts the user has already chatted with.

The chatbot dialog has a directed graph representation wherein vertices (or “nodes”) of various types hold the dialog content and other aspects of the chat logic, while edges (arcs between nodes) specify the available transitions. When a request is handled by the chatbot engine, this directed graph is traversed from the user’s current vertex until a vertex is reached that requires user input again. This procedure corresponds to a dialog turn. There is a range of vertex types in the dialog graph. Some vertices when visited invoke sending a message (text, attachment, etc) to the user, while others update a variable or assess a condition (and select an out-edge to traverse) based on user input or the value of a variable. It is common for the chatbot to return 2 or 3 responses to the user, which is an important way to break up longer pieces of dialog. A conversational humanness is conferred by a short thread sleep between dispatched responses as well as a “typing on” response to the user, which shows the chatbot is creating another message and appears as a dialog bubble with 3 moving dots.

The natural language understanding component of the system includes functionality for free-text intent classification. However, there are few locations within the dialog graph in which the chatbot encourages input of free text, with the dialog principally mediated by the provision of quick options for the user to choose from. When input is made by the user, free text is classified to 1 member of a predefined intent set through an application programming interface (API) request to DialogFlow [24]. Forty distinct intents were defined at a level sufficiently granular to discern expressions representing main mood/emotion states in a meaningful way. For example, intents were defined for anger, sadness, loneliness, stress, fear, anxiety, happiness, and excitement. At any vertex within the dialog graph where free text is handled, there are outgoing edges corresponding to specified intents and 1 fallback edge for free text that is unmapped. Multiple intents with similar characteristics will sometimes converge on the same child node when that node is sufficiently general to handle a group of intents (eg, fear, stress, and anxiety). For example, if a user typed “I’m sad” or “I’m down,” the chatbot responded with “Hey, I am sorry to hear you are feeling that way,” and then offered other resources.

Dialog authoring is undertaken using a web browser–based graphical user interface (GUI). The overall dialog has 1 entry point and is organized into dialog modules, such that a user moves through a sequence of modules, some of which are conditionally served. Given that the dialog is large, this modularization is particularly important to support nontechnical authors.

**Figure 2:**
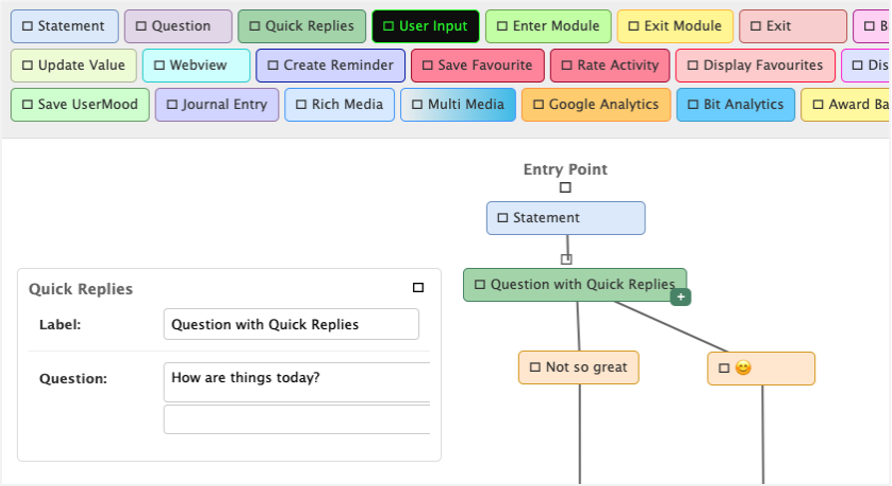
The dialog module authoring canvas of the chatbot architecture used for Aroha.

Figure 2 illustrates a portion of the user interface for dialog module authoring. Each dialog module has 1 entry point and 1 or more exit points (yellow “Exit Module” nodes). Authoring the dialog in each module involves defining both the sequence and content of dialog nodes. To define the sequence, the GUI canvas allows nodes to be dragged from the top toolbar into the canvas, and each out-edge must then connect (using a responsive snaplock) to a child node. Certain node types naturally allow for multiple out-edges. For example, a purple “Question” node receives free-text user input, and thus, the out-edges of this node type are associated with specific intents that free text is expected to be classified to (configured via a dropdown option available to this node type when clicked). The green “Quick Replies” node type also has multiple out-edges corresponding to a set of quick options a user may select from (beige colored labels to out-edges). There are also condition checking nodes (pink, “Branch”) that are transparent to the user but control the flow of the dialog using variables that can be set throughout the dialog (lime “Update Value” nodes).

The content of each node depends on the node type. Figure 2 illustrates editing of the content of a “Quick Replies” node. The content of this node type includes both the question presented to the user and a set of 1 or more quick reply options that the user may select from. “Rich Media” (light blue) nodes define an attachment response with an image or audio file, and thus, configuring content for this node type involves uploading the associated file type.

This authoring interface affords the opportunity for users without technical expertise to configure the dialog, and this helps to address the problem of the knowledge engineering bottleneck. Our experience with both Headstrong and Stress-Detox was that users without technical expertise can author the majority of dialogs for chatbots using this authoring system. For Aroha, clinical psychologists authored dialog modules directly.

The chatbot architecture was designed to interoperate with the HABITs information technology platform. To facilitate rapid and concurrent field trials within the HABITs project, a configurable web portal and suite of web services have been developed [25,26]. The portal supports online self-registration of users to trials with tailored project information and informed consent, as well as administration of online assessments at specified times. The web services allow applications to log event data (eg, sessions of use and activities completed) and link these with assessments for subsequent analysis. These features were used to set up the online trial of Aroha.

### Aroha Chatbot Development for Initial Rollout

In the context of a strict lockdown (all schools and nonessential workplaces closed, and the public instructed to stay at home and have contact only with those in their “bubble”), the expectation was that young people would be using Aroha at home, generally on their phones. The target age range for the chatbot users was 13 to 24 years.

Aroha was designed to have a more user-controlled flow than our previous chatbots that had a programmed set of day-by-day activities. After initial rapport building, including brief assessment and empathetic feedback, as well as psychoeducation around managing pandemic-related anxiety, the user was offered the option to select a module to suit them. Table 1 lists the modules in the initial release of Aroha and those added shortly after launch.

**Table 1:** Aroha chatbot modules.

Variable	Modules
First session/onboarding	Intro Onboarding Brief assessment Introductory information (modified at re-entry to just a greeting and repeat of the brief assessment)
Activity modules	Stay connected Calming activities Practice gratitude Spirituality Distract yourself Get active Get expert help General tips Self-care Have a routine Protect your sleep Alcohol and drugs
Activity modules added within 3 months after launch	Money worries Anger management Violence Prime Minister’s message
Outro	Recheck of the brief assessment Give feedback Check your hauora (health and well-being) Other resources Outro

Activity modules within Aroha were short. These modules teach evidence-based mental health strategies for good well-being that are based on best practice. Activities were designed to support young people to maintain general well-being within the context of the COVID-19 pandemic. The flow through the modules is largely driven by the user, and modules can be repeated, although users are encouraged to try another skill they might not have tried previously.

For example, the “practice gratitude” module promotes a grateful mindset and orients thinking toward positive things in life. The users are offered examples of things to be grateful for, such as whānau and technology, and then encouraged to make their own entry. Clinical trials show that practicing gratitude promotes happiness and well-being, and reduces symptoms of depression and anxiety [27]. Users could easily choose to start another module; otherwise, they were offered other resources upon module completion, as well as the option to finish the chat/exit.

**Figure 3:**
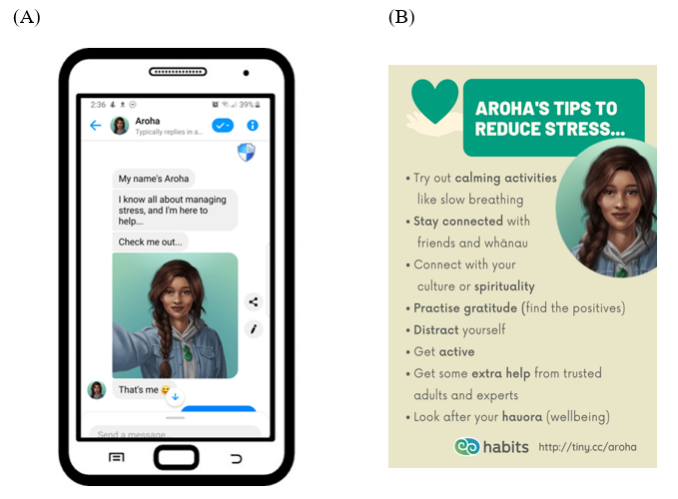
Example Aroha content. (A) Chatbot introduction with faux selfie. (B) “Poster” style advice.

Figure 3 illustrates some content from Aroha. All users had Aroha as a guide (unlike our previous chatbot deployments where there was a choice of 4 personas). Aroha offered an introduction and a “faux selfie” (Figure 3A) and reminded the user that the Aroha character is not a real person. The chat consisted of text and images in the message stream. “Poster” style content was frequently used to provide sets of related tips, such as “Aroha’s tips to reduce stress” (Figure 3B). Tips on a single image were able to be structurally linked, unlike a series of consecutive text bubbles. Posters were richly styled to reinforce the tone of the message, and users were offered the option to save posters to review later.

### Recruitment Procedure and Feedback

A tiny URL (tiny.cc/aroha) was created that redirected to the HABITs portal for Aroha trial enrollment and was used as the basis for disseminating the chatbot. The URL was used in communications released by the authors’ institution and interviews given by the authors, and was sent to contacts in the community inviting feedback and encouraging promotion of the service to young people. While these channels were unlikely to reach many young people directly, the intention was to reach professionals who would endorse the tool to young people, including guidance counsellors, mental health workers, teachers, and school senior management.

Early content generation was based on user testing (held online) with school guidance counsellors, clinicians, and rangatahi. We connected to our network of youth advisory groups and community organizations to recruit participants to a focus group that a clinical psychologist (SH) conducted in June 2020. The focus group was held online with 7 participants and had the specific objective of learning about the issues rangatahi faced with respect to the COVID-19 pandemic and lockdowns. The focus group consisted of introductions and karakia (Māori incantation and blessing), and an explanation of the Aroha chatbot, as well as the following guide questions: (1) What have your experiences and challenges been during COVID-19? (2) What do you expect the next 3 months to be like for you? (3) What are some of the challenges you or your friends might face going forward? (4) What do you expect the next week to be like? (5) What would the next 3 months ideally look like for you? (6) Do you think the challenges you’ve experienced over the last few months (referring back) will continue? The session was closed with karakia, and the youth voice from this focus group was used to inform the ongoing content iteration to the Aroha chatbot.

We conducted early user testing of Aroha using our existing networks and contacts. As we were working under lockdown conditions, feedback was collected from users remotely (using videoconferencing where possible) and in writing. We approached a small number of adolescents to try Aroha and asked them for their initial impressions, what they liked, and what improvements we could make. We also made Aroha available to an undergraduate class and invited students to send their feedback about the chatbot’s usability, style of communication, and features.

Given that the primary motivation for the development of Aroha was to ensure relevant and accessible support to young people, only a brief research assessment was incorporated via a single item assessing anxiety about COVID-19 on a scale with scores ranging from 0 to 10 (“no worries” to “totally freaking out”), collected at onboarding and the introduction of subsequent sessions. Users were invited but not required to record the single item at session outro. Those who used Aroha were also invited by automated email to give feedback about the experience of using Aroha 5 days after onboarding with the chatbot.

### Ethics Approval

This study was supported by an amendment to the University of Auckland Human Participants Ethics Committee protocol 023234 (a protocol initially used for Stress-Detox, amended to a lower target age, to provide COVID-19 focused content, and to remove the original study’s pre-post surveys). All participants provided informed consent.

## Results

### Initial Uptake

In the 2 weeks following the launch of the chatbot and the open trial, there were 393 registrations, and 238 users logged into the chatbot, of whom 127 were in the target age range (13-24 years). Moreover, 70.9% (90/127) were female and 47.2% (60/127) identified as New Zealand European. On average, target users engaged with Aroha for 11 minutes, and of the 127 users in the target age range, 31 returned for repeat sessions. There were 30 users (out of 81, 37%) who completed the pre-post measure of COVID-19 anxiety, both in the initial session and the outro, and these showed a reduction in self-reported anxiety from a mean of 5.1 (range 0-10; SD 2.6) to 4.3 (SD 2.5). Further uptake and evaluation data collected following the initial launch of the Aroha chatbot will be shared in a subsequent paper.

### Feedback

Feedback from users of Aroha who responded to the invitation sent by automated email has been provided in Textbox 1.

**Feedback quotes from young people in the target age range (13-24 years) who used Aroha.**

Question 1: “Did you find me useful?”

Responses:

1. “Yup I did, you gave a lot of good tips forma range of topics and it was rlly useful and good”
2. “Yes”
3. “Yes”
4. “Yeah, can I ask something personal lol”
5. “Yes, I did”
6. “Yes. Very good. Thank you”
7. “Yes, I did”

Question 2: “What was the most useful part?”

Responses:

1. “The range of tips from all the different topics”
2. “Ideas”
3. “The easy feeling of it being a conversation”
4. “Getting calm”
5. “Meditation”
6. “Breathing”
7. “Activities to reduce stress”

Question 3: “What could I do better?”

Responses:

1. “Ummm nothing really”
2. “Not sure”
3. “Some symbols don’t display on my phone, can make it hard to know what response I’m giving”
4. “Maybe ask what’s wrong first”
5. “I don’t know how to save favorites and I can’t see my progress”
6. “Nothing”
7. “Help with anxiety”

Feedback highlighted that Aroha was accessible and acceptable. Young people gave positive feedback about the aspects of engagement that were specifically used to encourage uptake. User comments were generally encouraging and highlighted the need for this intervention at the time of global uncertainty and high stress in the community (Textbox 2).

**Feedback (illustrative quotes) from young people and community members on the COVID-19 pandemic lockdown and experience of Aroha.**

**COVID-19 pandemic and lockdown feedback**

“The struggle is real”

“Thought I’d miss my friends but I didn’t”

“Hard not to take them home and have a tangi [mourning ceremony]”

“Not being able to grieve in a traditional way affected my mental health”

“I love them to death but they were really annoying and especially with my uni work…having to home school my 4 year old sister….”

“All my 8 siblings being at home”

“Guilt of not using my time in lockdown in the most productive way”

“Will New Zealand get attacked too by the corona virus”

**Aroha chatbot feedback**

“I found it cool to see messenger used”

“It felt like you were actually having a casual conversation with someone else rather than being lectured to”

“the use of emojis and GIFs helped to increase my engagement”

“I really liked how Aroha used language that I use to communicate; she used words like *heaps* and *whānau* which made it feel like she was down-to-earth and easy to engage with and talk to”

“Having it set within messenger makes it feel natural as if you were talking to one of your friends and it normalizes the idea of talking/reaching out to somebody”

“I liked that you could respond with a pre-determined emoji as it took the pressure off trying to think of a response. She was very clear and easy to understand and the replies were also made very easy”

**Community feedback**

“This chatbot is neat! Aroha breathed positivity; I can imagine this helping many going through a tough time during this pandemic”

“Having the chatbot sit within Messenger (and existing app that users already had on their phones) made it easy to access and made getting help easier”

“Young people liked the language, use of humor, emojis, GIFs and felt that all those features *humanized* Aroha”

“The dialogue was praised for being realistic and the avatar was well liked, and many people felt a sense of connection to it”

“There were concerns about a limited range of responses, and some wished there was more opportunity for the user to express their emotions through free text”

“I’m not sure how you would develop such a software, but it would also be great to engage with a more empathetic figure, someone that can understand emotive language coming through text language”

Feedback also highlighted important improvements that were needed to increase accessibility and engagement. The themes were consistent across respondents (n=20) and were thematically grouped and included the following: (1) emergent needs as the COVID-19 situation evolved (in Aotearoa, the initial fear of contracting COVID-19 quickly subsided due to strict social restriction measures instituted by the government, and more salient concerns evolved, such as unemployment and resulting poverty, as well as experiencing violence and abuse while in lockdown); (2) improved conversationality; and (3) improved personalization of content. It was further deemed important to adapt the dialog content to reflect the changing nature of the pandemic.

The development effort in response to initial feedback included expanding the set of dialog modules to include content for emergent needs. Modules were developed to address financial stress and domestic violence. Having a large amount of informational content requires presenting this content in diverse ways to foster user engagement. In the months following the initial launch, we expanded on the use of posters (image files) within the messenger channel by developing more dynamic webviews (single webpages using JavaScript for dynamics) to deliver content. This included a public health message from the Prime Minister’s Office, and there was iteration through each statement of the message (describing personal top tips for managing stress) in sequence on a touch event.

The need for improved conversationality and personalization was addressed by (1) enlarging the set of defined intents, (2) making some intent areas more fine-grained, (3) enlarging the set of out-edges (locally recognized intents) at free-text question nodes, and (4) implementing “priority intents” (intents that are detected by the dialog agent independent of a user’s location within the overall dialog). Prior to implementing priority intents, except for risk phrase detection for statements representing potential self-harm expressions, free-text user input that did not match any intent-labeled out-edge at a free-text or quick reply question node would invoke only a general response to the default fallback out-edge. For example, if a user expressed that they were feeling *nervous* at a dialog node with labeled out-edges for sadness, happiness, or default, then the chatbot would respond only with a statement that was generally applicable such as an emoji “shrug” or “ponder.” This impairs the chatbot’s pretense of intelligence and conversationality and thus user experience. “Priority intents” have since been defined that allow the chatbot to detect a set of expressions even if they are not locally configured and thus allow the user to digress elsewhere in the dialog. These priority intents include expressions related to self-harm, risk of abuse, low mood, fear, boredom, and quit/exit. This change shifts the system toward being more responsive to the user’s agenda as compared to its preprogrammed agenda. User-driven digressions made possible by priority intents are logged as usage data on the HABITs platform to elucidate the frequency of priority intent activation.

Events following the initial launch have underscored the need for easy content modification that can quickly be rolled through to the production chatbot service. For instance, in August 2020, there was a rapid reintroduction of COVID-19 restrictions in Aotearoa, but this time, there was strong regional variation (with the outbreak being focused in Auckland). Similarly, there were many regional changes during 2021, with varying restriction levels of stay-at-home orders from August 17, 2021, to December 2021 [28]. This required adjustment of both the tone of content and asking about user lockdown conditions, such that advice was relevant to local conditions.

## Discussion

### Principal Findings

Our experience of developing the Aroha chatbot indicates the feasibility of implementing chatbot-based digital mental health support in response to emerging events. The straightforward authoring that is possible through the content management system and the chatbot architecture means that mental health and well-being support can be tailored to any event, not only that of a global pandemic.

Initial user feedback provided guidance to update content and features as the COVID-19 situation in Aotearoa evolved. In the case of Aroha, we initially used techniques drawn from CBT and positive psychology, and integrated in a bicultural context, which have been shown to be efficacious across many groups and via conversational agents [12,29]. Based on user feedback, we identified the need for additional content, particularly in the areas of distress related to problems of living. Feedback also gave a clear signal that users wanted more dynamic conversationality. Initial efforts had been focused on imparting a large amount of dialog content into Aroha to cover the variety of different stressors that users were facing given the pandemic context. A strength of our chatbot architecture is the ability to easily author large amounts of dialog content, including directly by domain experts. Yet, user feedback established that Aroha required better ability for a user to drive the conversation rather than always follow the chatbot-guided dialog.

In a viewpoint article, McGreevey et al identified a range of considerations for implementing conversational agents in health care [30]. Among the leading considerations were patient safety and trust, and transparency. A key element of Aroha is the use of evidence-based approaches with expert authorship of content. A further element is that the agent persona, although drawn as a relatable human, clearly identifies itself as a computer program. Further, detection of self-harm phrases in user input at any time triggers escalation to confirmation, expression of empathy, direction to a help hotline, and shut down of the computer-based dialog.

Another important consideration is health equity. In Aotearoa, it is crucial for the chatbot to at least be bicultural and show positive effects for Māori. To highlight this and to ensure a culturally responsive product, we developed a chatbot with a Māori persona that had a background story about how it was designed. We also ensured bicultural clinician input and authorship, which ensured that there was relevant Māori content available in the chatbot. While anecdotally it appears that these values were reflected in good reach for rangatahi Māori, further analyses and discussions will be presented in a subsequent paper.

In terms of cybersecurity, extensive user input is not encouraged (which, at any rate, we would not be able to use therapeutically in a safe and trustworthy manner with current technology). Further, specific user input is not logged to our research platform database (only the series of activities and intents to understand usage). However, the conversation log is held with Facebook, and while the project information presented in the user consent process says that the interaction with the chatbot is visible to Facebook and is subject to Facebook’s privacy policy, most young people would not read the information in full or entirely appreciate such a notice. In terms of research, development, and innovation considerations, we have framed Aroha as a trial with informed consent, and we believe this is appropriate until the effectiveness of the intervention is better understood.

### Limitations

This study has some limitations. We have not yet conducted a randomized controlled trial of Aroha [31]. In fact, the initial rollout has emphasized ease of user experience over data collection. However, the intervention is based on evidence-based therapies, and indeed the context of COVID-19–induced stress is sufficiently dynamic that any trial will have limitations in terms of transferability.

We included a research assessment via a single-item anxiety question (0-10 scale, “no worries” to “totally freaking out”). A single-item measure of anxiety limited our ability to assess severity, although single-item measures have shown reasonable sensitivity and specificity in screening for anxiety in the hospital setting [32]. The advantage is that they are quick and simple to implement in a chatbot application, but further validation of this is necessary.

In the months following launch, we received funding for further structured user engagement (youth focus groups) and implementation of a Te Reo Māori version. The natural language functionality is being enhanced by including free-text named entity recognition. As a longer-term direction, we are exploring how deep learning could be used to create a more dynamic and engaging user experience. The ability to create empathetic dialog and achieve large numbers of conversational turns per session as demonstrated with Xiaoice [33] would be beneficial to our system if only to lead users to a larger “dose” of chatbot-based therapy. However, we would not want to use deep learning in a way that compromises the quality of expert-authored content. In the first instance, our expanding use of intent recognition provides a compromise that preserves the use of expert-authored content in the chatbot response. A further extension of deep learning that is consistent with our approach would be deep learning of dialog policy (eg, machine learning of what module to choose or recommend next, but where the module content is still expert authored [34]). An additional option is to allow machine-learnt dialog for rapport-building chit-chat and possibly to learn more about the users and their needs, but then fall back to more reliable content for therapeutic advice.

### Future Directions

One area of further work that we have pursued is the development of a custom chat app as an alternative to Messenger. There are benefits of using Messenger, such as capitalizing on the fact that young people habitually use social media and many already use Messenger [35,36], and that there is no requirement to download an app to use the chatbot. However, development of a standalone app means that a superior experience can be delivered without limitations on user notifications or logging of conversation details with a third party.

Since its initial launch, the Aroha chatbot has evolved from the initial main objective of supporting young people through COVID-19 lockdown and strict stay-at-home orders. Aroha has developed to include general mental health and well-being support. For example, content for Matariki (Māori new year) and Kohinga Māori activities (a collection) were added to the chatbot, and modules for current events like daylight savings and examinations were included. Supporting mental well-being is a notion in line with Te Kāwanatanga o Aotearoa (the New Zealand government’s) Kia Kaha, Kia Māia, Kia Ora Aotearoa, and COVID-19 psychosocial and mental well-being plan. This initiative aims to support individuals, whānau, and communities to respond, recover, adapt, and thrive in the context of the COVID-19 pandemic [16]. Following the Kia Kaha plan (released in 2020), a long-term plan, Kia Manawanui: Long-term pathway to mental well-being, was published in 2021 [17]. Kia Manawanui outlines the transformation of the approach to mental well-being. The Aroha chatbot has and continues to be an imperative component of psychosocial recovery for rangatahi.

### Conclusions

We have identified elements of a chatbot architecture sufficient for responsiveness to emerging situations, such as a pandemic lockdown, with easy authoring by domain experts and a rapid deployment channel as cornerstones. In our case, the deployment channel included a configurable portal for web-based trial recruitment linked to chatbot interaction through Facebook Messenger. Further, we found user demand for increased responsiveness to a range of inputs from boredom to fear, as well as a need for additional content. This emphasized the need for a flexible and extensible output, and the ability to easily update the service. Our experience is that an architecture with these elements for creating supportive chatbots has wide application for flexible and rapid responsiveness to other events and situations.

## Abbreviations

CBT: cognitive behavioral therapy
GUI: graphical user interface
HABITs: Health Advances through Behavioural Intervention Technologies
SPARX: Smart, Positive, Active, Realistic, X-factor thoughts
